# Depth-Camera-Based Under-Blanket Sleep Posture Classification Using Anatomical Landmark-Guided Deep Learning Model

**DOI:** 10.3390/ijerph192013491

**Published:** 2022-10-18

**Authors:** Andy Yiu-Chau Tam, Li-Wen Zha, Bryan Pak-Hei So, Derek Ka-Hei Lai, Ye-Jiao Mao, Hyo-Jung Lim, Duo Wai-Chi Wong, James Chung-Wai Cheung

**Affiliations:** 1 Department of Biomedical Engineering, Faculty of Engineering, The Hong Kong Polytechnic University, Hong Kong 999077, China; 2 Department of Bioengineering, Imperial College, London SW7 2AZ, UK; 3 Research Institute of Smart Ageing, The Hong Kong Polytechnic University, Hong Kong 999077, China

**Keywords:** sleep posture recognition, sleep surveillance, sleep monitoring, sleep behavior, ubiquitous health, digital health

## Abstract

Emerging sleep health technologies will have an impact on monitoring patients with sleep disorders. This study proposes a new deep learning model architecture that improves the under-blanket sleep posture classification accuracy by leveraging the anatomical landmark feature through an attention strategy. The system used an integrated visible light and depth camera. Deep learning models (ResNet-34, EfficientNet B4, and ECA-Net50) were trained using depth images. We compared the models with and without an anatomical landmark coordinate input generated with an open-source pose estimation model using visible image data. We recruited 120 participants to perform seven major sleep postures, namely, the supine posture, prone postures with the head turned left and right, left- and right-sided log postures, and left- and right-sided fetal postures under four blanket conditions, including no blanket, thin, medium, and thick. A data augmentation technique was applied to the blanket conditions. The data were sliced at an 8:2 training-to-testing ratio. The results showed that ECA-Net50 produced the best classification results. Incorporating the anatomical landmark features increased the F1 score of ECA-Net50 from 87.4% to 92.2%. Our findings also suggested that the classification performances of deep learning models guided with features of anatomical landmarks were less affected by the interference of blanket conditions.

## 1. Introduction

Sleep is an indispensable activity that maintains physiological functions and processes in life [1]. Humans spend one-third of their lifetime sleeping [2]. Adults are recommended to sleep at least seven hours a day to mitigate the risks of obesity, diabetes, hypertension, cardiovascular disease, stroke, mental distress, and premature death [3]. Poor sleep quality not only affects productivity and academic performance, but may also lead to injury and car accidents [4]. Sleep postures are closely related to sleep quality and disorders [5], and an analysis of sleep posture changes have been used to identify the deep sleep stage [6]. It was reported that individuals with poor sleep quality spent more time in provocative poses with more frequent posture changes [5]. While sleep aims to relieve the fatigue of muscles and ligaments, a side-lying posture accompanied by poor support may lead to a sagging spine and sinking lumbar, resulting in sleep-related musculoskeletal problems, such as neck and back pain [7,8,9]. Sleep postures and posture changes were also associated with sleep bruxism [10], carpal tunnel syndrome [11], and restless leg syndrome [12]. Additionally, for patients with obstructive sleep apnea, supine and prone postures may affect their respiration because of tongue and palate prolapse and pressure on the thorax [13]. Investigating sleeping postures is important to understand the pathomechanism and design interventions for sleeping disorders [14], while different sleep assessment technologies, such as polysomnography, unobstructive intelligent sensors, and wearables, could monitor sleep behaviors and physiological signs for the better diagnosis of sleep problems [15].

Clinically, to evaluate sleep disorders, individuals need to stay overnight in a specialized clinic for a sleep test. Not only is the process expensive, annoying, and requires medical and technical professionals to operate, but the unfamiliar sleeping environment can affect the test’s accuracy. Ubiquitous sleep posture monitoring is, thus, necessary for the long-term assessment of sleep deprivation and sleep disorders in a nonclinical setting [15].

Traditional sleeping posture measurements include videotaping with manual labeling or self-reported assessments, which can be tedious and inaccurate [16,17]. Recently, different nonintrusive technologies have been used in sleeping posture recognition, including visible light, infrared, and depth cameras [18,19], inertia measurement units with a wireless connection [20], radar/radio sensors [21,22,23], and pressure sensors [24,25,26]. However, there have been few attempts to recognize and classify sleeping postures accurately [27]. Various machine-learning-based techniques have emerged recently, including learning, the support vector machine (SVM), k-nearest neighbors (KNNs), and a convolutional neural network (CNN), which aim to improve the accuracy of posture classification for both optical and pressure sensing [18,19,28,29,30,31,32,33].

Depth-camera-based systems are preferred for sleep monitoring, since they are noncontact, easier to maintain, allow for privacy, and can work well at nighttime without visible light [34]. Grimm, et al. [35] classified three common sleeping postures (supine and left-, and right-sided lying) using a convolutional neural network (CNN) with a good overall accuracy of 97.5% [35]. Similarly, Ren, et al. [36] reported high accuracy in classifying seven postures using a support vector machine (SVM) from images acquired using a Kinect Artec scanner. Nevertheless, these systems did not practically consider the interference of blankets, which is known as the primary barrier for noncontact sleep surveillance [37]. Our previous study classified seven postures under different thicknesses of blanket conditions using a CNN, but did not achieve an adequately satisfactory accuracy (<90%).

In summary, wearable sensors can reach high accuracy in posture classification, but are uncomfortable and comply poorly [38]. Additionally, blankets challenge the classification accuracy of noncontact optical systems, in addition to causing difficulty in distinguishing prone from supine postures [37]. To this end, we propose to enhance the accuracy of the under-blanket sleep posture classification with a new deep learning model architecture with a guided input of anatomical features generated using a pose estimator [39]. The classification covers seven common sleep postures, including the supine posture, prone postures with the head turned left and right, left- and right-sided log postures, and left- and right-sided fetal postures. The key contributions of this study are as follows:

- We developed a posture classification system that can be generalized to various blanket conditions.
- We proposed an integrative innovation for the deep learning model to improve the classification performance through anatomical landmark features generated using a pose estimator.

## 2. Materials and Methods

### 2.1. Participant Recruitment

We recruited 120 healthy adults (61 male and 59 female) in this study from the university and community. Their mean age was 45.5 years (SD: 20.7; range 11 to 77). Their average height and weight were 165 cm (SD: 9.02; range 148 to 185) and 60.6 kg (SD; 11.2, range 40.8 to 98.1), respectively. The age distribution in different genders is shown in Table 1. We endeavored to recruit participants of different ages to adequately test the robustness of the deep learning model. Participants were excluded if they reported severe sleep deprivation, a sleep behavioral disorder, musculoskeletal pain, or a deformity.

The experiment was approved by the Institutional Review Board (reference number: HSEARS20210127007). All participants signed an informed consent form after receiving oral and written descriptions of the experimental procedures before the start of the experiment.

### 2.2. Hardware Setup

We used an active infrared stereo technology depth camera (Realsense D435i, Intel Corp., Santa Clara, CA, USA), which incorporates an auxiliary visible light RGB camera and inertia measurement unit (IMU). The depth camera had a resolution of 848 × 480 pixels, sampled at six frames per second. The camera was mounted 1.6 m above the bed, which was 196 cm long, 90 cm wide, and 55 cm tall. During the experiment, we considered the no-blanket condition and three thicknesses of blankets (thick, medium, and thin) to resemble real life scenarios. Respectively, the blankets were the FJALLARNIKA extra warm duvet (8 cm thick), SAFFERO light warm duvet (2 cm thick), and VALLKRASSING duvet (0.4 cm thick). All of them were sourced from IKEA (Delft, The Netherlands).

### 2.3. Experimental Procedure

**Figure 1:**
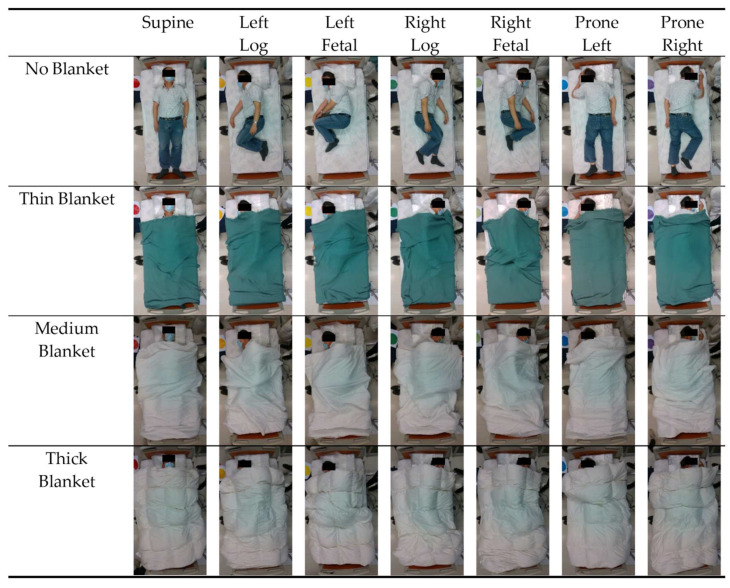
Seven sleep postures classified in the deep learning models: supine, left-sided log, left-sided fetal, right-sided log, right-sided fetal, prone with head turned left, and prone with head turned right.

During the experiment, participants were instructed to lie on the bed in seven sleep (recumbent) postures in order: (1) supine, (2) prone with head turned left, (3) prone with head turned right, (4) log left, (5) log right, (6) fetal left, and (7) fetal right (Figure 1). Time was given to allow participants to adjust to their most comfortable position before data collection.

During the data collection for each posture, the participants maintained their posture. Then, we covered the participants with blankets in the sequence of thick, medium, thin, and no blanket over their full body (except head). Data were collected continuously throughout the experiment and postures were labeled by placing a color-coded paper adjacent to the bed.

### 2.4. Data Processing

The RealSense Software (Intel, Santa Clara, CA, USA) Development Kit was utilized for extracting the raw data. A pair of synchronized RGB and infrared depth image data was extracted for every posture and quilt conditions. For every subject, 28 pairs (4 quilt conditions × 7 classes) of images were captured, resulting in a total of 3360 pairs of images. Additional labeling and cropping were performed through a manual pipeline program written using OpenCV SDK (Intel, Santa Clara, CA, USA). Pairs of images were labeled based on the color-coded paper and cross-checked by observing the video, and were cropped to combine the pairs of images to the bed area.

A customized data augmentation strategy was applied to generate more blanket conditions (blanket-augmented dataset), described previously [14]. In brief, data augmentation was conducted using the affine transformation of the depth image data and the data fusion of the blanket conditions. It shall be noted that the data augmentation took place and was incorporated inside the model training process.

**Figure 2:**
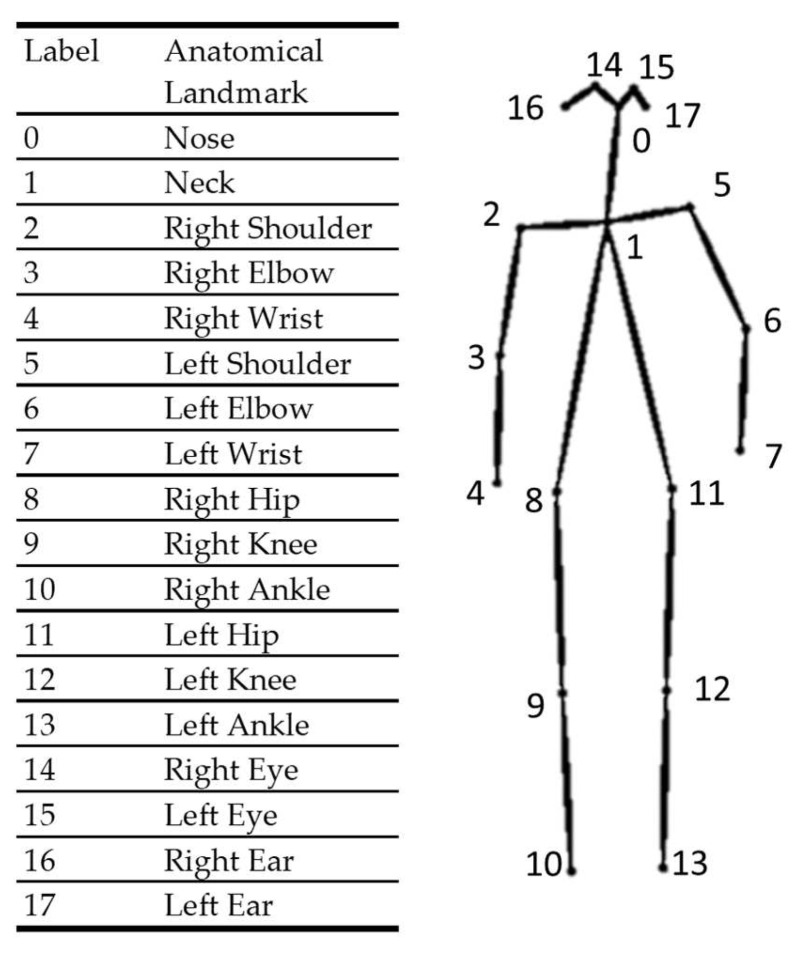
The anatomical landmarks generated by the pose estimator (OpenPose).

The RGB images collected from the auxiliary visible light camera were input into an open-source deep-learning-based human pose estimator. The key role of the human pose estimator was to provide anatomical landmark information to guide the deep learning model. We utilized OpenPose [39] as the human pose estimator. OpenPose adopts a strategy named the part affinity field, which is a nonparametric representation of association used to encode body part positions and orientations [39]. The estimator learned the part detection and association simultaneously, and identified the poses through a greedy parsing algorithm [39]. A map of confidence levels was calculated to represent the probability that a particular body landmark could be detected in any given pixels. The two-dimensional coordinates of a particular body landmark were taken as that of the maximum of the confidence map. Subsequently, the pose estimator could output ordered pairs of two-dimensional coordinates of 18 anatomical landmarks (detailed in Figure 2) and confidence level values. We then mapped the coordinates to corresponding depth images.

### 2.5. Model Architecture

**Figure 3:**
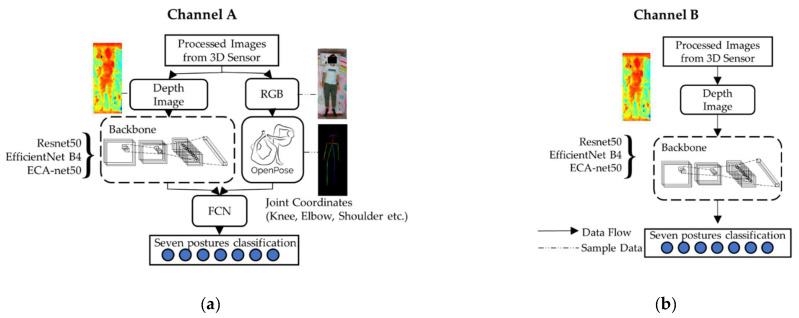
Model training architectures (system units) with the ResNet-34, EfficientNet B4, and ECA-Net50 backbones: (**a**) Channel A: with pose estimator; (**b**) Channel B: without pose estimator.

We exploited six model architectures, which were three deep learning models (ResNet-34, EfficientNet B4, and ECA-Net50), trained using depth camera images, with (Channel A) and without (Channel B) pose estimator input, as shown in Figure 3 [39]. With regard to the deep learning models, in brief, ResNet (short for residual network) [40] employed a residual network architecture and solved the vanishing gradient problem using traditional deep neural learning by skipping some layers (shortcut connection). EfficientNet [41] utilized a compound scaling method that sedulously scaled the network depth, width, and resolution, and was proven to outperform traditional CNNs with arbitrary and uniform network dimensions. ECA-Net (short for efficient channel attention module for deep CNNs) [42] is a lightweight model that aimed to avoid channel dimensionality reduction, but maintained the consideration of cross-channel interactions through a fast 1D convolution with a kernel predetermined using channel dimensions.

As shown in Channel B (Figure 3b), for the architecture without the pose estimator, the depth image data were input into the backbone of the model. The model then output the classified postures.

For the architecture with the pose estimator (Channel A, Figure 3a), the RGB image data were input into the pose estimator to approximate the coordinates of anatomical landmarks. The depth image data were input into the backbone of the model. Thereafter, the coordinate information was concatenated with the output of the backbone’s fully connected layer into another fully connected layer for posture classification.

### 2.6. Model Training

Overall, the models were trained with the depth image data. Moreover, the model training in Channel A contained additional information on the coordinates of anatomical landmarks, which was generated using the pose estimator (OpenPose) over the no-blanket dataset, and mapped on the blanket dataset correspondingly (i.e., data mapped on the same posture of the same participant). Channel B did not have the coordinate information for model training. The process of Channel A and B selection was detailed in Section 2.7.

We implemented a data slicing strategy at an 8:2 training-to-testing ratio (i.e., 96 participants with 2688 images for model training). Model training was implemented using the PyTorch Deep Learning Framework 1.10 [43]. A randomized grid search (*n* = 10) on hyperparameters (learning rate and L2 regularization) was implemented over values of 1 × 10^−3^, 1 × 10^−4^, 1 × 10^−5^, 1 × 10^−6^, 2 × 10^−3^, 2 × 10^−4^, 2 × 10^−5^, 2 × 10^−6^, 5 × 10^−3^, 5 × 10^−4^, 5 × 10^−5^, and 5 × 10^−6^. Cross-entropy loss was used as the objective function for the model training. Afterwards, the Adam optimizer was set with a fixed learning rate of 0.0001, L2 regularization of 0.0005, and a batch size of eight. The batch size of eight was predetermined due to the constraint of computational power. Based on the learning curve, we decided to train the model with 1000 epochs.

### 2.7. System Architecture (with Model Testing)

**Figure 4:**
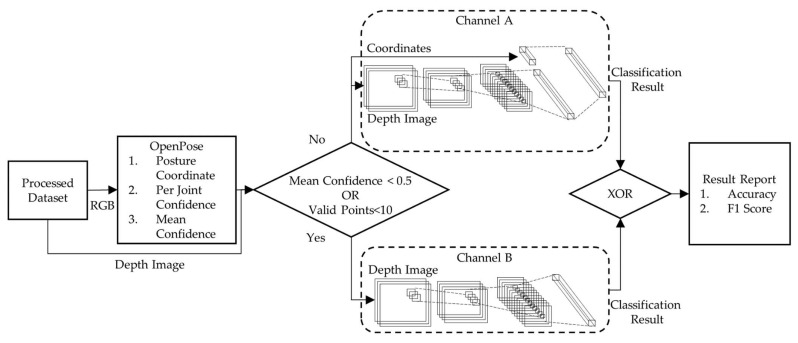
System application architecture to predict sleep posture from testing data after the models were trained.

Figure 4 shows the system level after the models were trained. The test data were input into the pose estimator (OpenPose) to generate point coordinate information and confidence levels. The selection of Channel A or B for a particular piece of data sample was determined with the confidence level of the coordinate points generated using the pose estimator. If the confidence level of the pose estimation averaged over all point coordinates was less than 0.5, or the number of valid points (i.e., NULL confidence level) was less than 10, the data were delivered to Channel B without a pose estimation, shown in Figure 4. Otherwise, the data headed to Channel A with a pose estimation. The final prediction was determined with an exclusive OR (XOR) of outputs from Channels A and B (Figure 4).

### 2.8. Evaluation

The models were evaluated using accuracy and the F1 score, which corresponded to the proportion of correct predictions and the harmonic mean of recall and precision [44]. The primary evaluation was conducted to compare the accuracy of the three deep learning models with and without guidance from anatomical landmarks. The secondary evaluation was conducted to compare the performance of the models trained with different blanket datasets. For models without anatomical landmark guidelines, all data flowed to Channel B only. For models with anatomical landmark guidelines, since some data may or may not have fulfilled the confidence level criteria, data could flow in either Channel A or B.

## 3. Results

### 3.1. Performance of Different Models with and without Pose Estimator

The outcome measures of accuracy and F1 score are presented in Table 2. In general, models guided with anatomical features had a higher accuracy and F1 score. The mean accuracy and F1 score of the models without anatomical feature guidance were 86.6% and 86.1%, whereas those with the pose estimator were 90.8% and 91.4%. The elevation was approximately 2% to 7% and 3% to 4%, respectively, for accuracy and the F1 score. ECA-Net demonstrated the best performance with an accuracy of 91.5% and an F1 score of 92.2%.

### 3.2. Influence of Blankets on Model Performance

The accuracy and F1 score of the models with the pose estimator trained using different blanket conditions are presented in Table 3. Similarly, ECA-Net50 seemed to have superior performance overall. Nevertheless, EfficientsNetB4 outperformed the others for the all-blanket dataset. In general, the blanket conditions did not change the classification accuracy considerably for all models enhanced with the pose estimator, given that the deviation was approximately 2%. The classification accuracy was less affected by the blanket conditions when the pose estimator was present.

## 4. Discussion

This study endeavored to improve the classification performance of sleep postures with blanket interference using the input and guidance of anatomical information from a pose estimator. The significance of this study laid in the development of a relatively accurate posture classifier of under-blanket sleep postures, and, thus, to facilitate the monitoring and diagnosis of sleep disorders. Key findings of this study included:

- ECA-Net50 produced the best classification results with an F1 score of 87.4%.
- The performance of ECA-Net50 was improved by the anatomical landmark feature from 87.4% to 92.2%.
- Classification performances of ECA-Net50 with anatomical landmark features were less affected by the interference of blanket conditions.

Sleep postures were featured by the location of the head–trunk and the placement of the limbs. Therefore, anatomical landmarks or musculoskeletal joint positions were intuitively critical features representing the sleeping postures. Furthermore, the deep learning models were less sensitive to the interference of blanket conditions after guidance from anatomical landmarks because of the identification of critical anatomical features. In particular, the level of improvements was considerable in ResNet and ECA-Net, with an increment of more than 7% and 4%, respectively. We evaluated that the anatomical landmark-guided strategy was more significant to models that required larger datasets. Moreover, ECA-Net produced the best accuracy among the three models. The reason could be that the model applied an “attention” function to leverage the weights of anatomical landmark features, which were determinants of sleep postures, resulting in improved accuracy. However, it shall be noted that the current pose estimator relied on the input of RGB images, which could not be acquired at night without lights. The further development of the pose estimator to accommodate depth camera images is necessary.

Apart from noncontact methods, other modalities are commonly used for sleep posture classification. Ostadabbas, et al. [45] developed a body pressure measurement system to classify three sleep postures (supine and left- and right-sided lying) using the Gaussian mixture model with an overall accuracy of 98.4%. Fallmann, et al. [38] mounted accelerometers onto the trunk and limbs of participants in an attempt to classify six sleep postures using the matrix learning vector quantization approach. Despite the higher accuracy, these noncontact methods can be obstructive, difficult to maintain, and poorly complied, which affects the feasibility of long-term surveillance, particularly in the clinical setting [46].

There was one study considering sleep posture classification with blanket conditions using a noncontact approach. Mohammadi, et al. [47] utilized the Microsoft Kinect infrared depth camera to classify 12 sleep postures with thin blankets and achieved an accuracy of 76%. Our study demonstrated a higher accuracy with different thicknesses of blankets. In addition, the data augmentation subsumed hypothetical blanket variations that enhanced the generalizability.

In fact, a four-posture classification system (i.e., supine, prone, right, and left lateral) was commonly adopted [19,48,49,50,51] because of its relationship with the Apnea-Hypopnea Index (AHI) [37,52]. Some studies excluded or merged prone posture with supine posture due to the difficulty in classifying them [53,54,55,56,57,58]. A higher granularity classification was of particular interest, especially subdividing the lateral posture into log and fetal postures [59,60]. We found one study exploring the yearner posture, but ignoring the log and prone postures [59]. In this study, we classified seven postures that involved a fine granularity on the prone posture of head positions (left and right) and lateral postures (log and fetal), which covered the majority of common sleep postures. Additionally, finer granularity classifications were also endeavored on the location of the limb placement [38,47,61,62] or joint angles [63,64] to address sleep-related musculoskeletal disorders [37]. Furthermore, while existing finer granularity classifications often exploit head or limb orientation/placement, the postures of the head, trunk, and limbs can be classified separately, since some sleep disorders are sensitive to postures over individual body parts. For example, a supine head posture can worsen the conditions of sleep apnea, but not the trunk [37,52]. A hierarchical posture classification system with an analysis of localized postures of body parts to generalized sleep postures could be beneficial in the clinical diagnosis of sleep disorders.

It is often a challenging issue to determine the sample size for machine learning models. Machine learning models can suffer from overfitting, underfitting, nonconvergence, and/or bias in accuracy estimates if there is a scarcity of samples [65,66]. Data augmentation has been widely accepted as a technique that ameliorates the sample size issue in machine learning [65,66]. In our study, we endeavored to synthesize more blanket conditions using a data augmentation technique [14], which produced a comparable data size per class as a common benchmark dataset, such as the CIFAR-100 dataset [67].

There were some limitations in the study. First of all, the pose estimator may not have always successfully produced valid coordinates for the anatomical landmarks. The guided strategy was bypassed for those challenging cases in the model training process despite a satisfactory rate of accuracy being possible to achieve. Secondly, the evaluation of our current system targeted discrete events (i.e., based on static images). We are currently heading towards incorporating the model into a video-based system to monitor the proportion of different postures during the sleep course. Thirdly, we recruited normal/healthy subjects only through convenient sampling that could have led to some level of imbalanced age and gender distributions. Since our model, at this stage, only considered posture classifications for normal individuals, we did not consider including participants with sleep disorders. Finally, the deep learning model was a totally unsupervised feature discovery model used alongside a classifier. The model may or may not have utilized the anatomical features, though it might have been critically weighed because of its strong association with sleep postures. Nevertheless, deep learning models are often referred as an inscrutable blackbox models. The methodology could not obtain the physical meaning and relationship between features, which could be important for clinicians [68]. Future studies should consider to quantify the spine curvature and joint kinematics to better understand and monitor sleep-related musculoskeletal disorders [7].

## 5. Conclusions

This study presented a new deep learning model architecture for an under-blanket sleep posture classification system using infrared depth camera images. A feature of the model’s architecture included the utilization of a pose estimator to generate anatomical landmarks to guide the deep learning models, which improved the posture classification accuracy and benefitted pragmatic applications over generalized blanket scenarios in real-life. The F1 score of ECA-Net50 increased from 87.4% to 92.2% when anatomical landmark information was considered using the pose estimator. ECA-Net50 adopted an “attention” strategy that might have leveraged the weights of the critical determinants of sleep postures (i.e., the anatomical landmark information). In addition, our findings also supported that the performance of deep learning models enhanced with anatomical landmark was less affected by the interference of blanket conditions.

## Figures and Tables

**Table 1:** Histogram on the age and gender distribution of participants.

Characteristics		Number of Participants (*n* = 120)	Proportion (%)
Gender	Male	61	50.8
	Female	59	49.2
Age Group	18–19	10	8.3
	20–29	35	29.2
	30–39	11	9.2
	40–49	3	2.5
	50–59	11	9.2
	60–69	37	30.8
	70–79	13	10.8

**Table 2:** Performance of deep learning models (EfficientNetB4, ResNet50, and ECA-Net50) trained using augmented dataset with and without pose estimator.

Outcome	Channel	Pose Estimator	Deep Learning Models		
			EfficientNetB4	ResNet50	ECA-Net50
Accuracy	A + B	Yes	89.7%	91.1%	91.5%
	B	No	87.4%	83.6%	88.7%
F1-score	A + B	Yes	90.8%	91.3%	92.2%
	B	No	87.3%	83.6%	87.4%

**Table 3:** Performance of deep learning models (EfficientNetB4, ResNet50, and ECA-Net50) with pose estimator input trained using datasets with no blanket, all blankets, and augmented data.

Outcome	Dataset	Deep Learning Models with Pose Estimator		
		EfficientNetB4	ResNet50	ECA-Net50
Accuracy	No blanket data	91.1%	91.7%	92.3%
	All blanket data	91.4%	89.3%	90.9%
	Augmented data	89.7%	91.1%	91.5%
F1-score	No blanket data	91.1%	91.6%	92.2%
	All blanket data	91.9%	88.9%	91.7%
	Augmented data	90.8%	91.3%	92.2%

## Data Availability

The data presented in this study are available on request from the corresponding author.
